# Association of *SULT1A2* rs1059491 with obesity and dyslipidaemia in southern Chinese adults

**DOI:** 10.1038/s41598-023-34296-4

**Published:** 2023-05-04

**Authors:** Hai-Yan Lv, Guifeng Shi, Cai Li, Ya-Fei Ye, Ya-Hong Chen, Li-Hua Chen, Tao-Hsin Tung, Meixian Zhang

**Affiliations:** 1 Department of Clinical Laboratory, Taizhou Hospital of Zhejiang Province Affiliated to Wenzhou Medical University, Linhai, 317000 Zhejiang China; 2 Department of Preventive Health Care, Taizhou Hospital of Zhejiang Province Affiliated to Wenzhou Medical University, Linhai, 317000 Zhejiang China; 3 Department of Neurology, Taizhou Hospital of Zhejiang Province Affiliated to Wenzhou Medical University, Linhai, 317000 Zhejiang China; 4 Health Management Centre, Taizhou Hospital of Zhejiang Province Affiliated to Wenzhou Medical University, Linhai, 317000 Zhejiang China; 5 Public Scientific Research Platform, Taizhou Hospital of Zhejiang Province Affiliated To Wenzhou Medical University, Linhai, 317000 Zhejiang China; 6 Evidence-Based Medicine Center, Taizhou Hospital of Zhejiang Province Affiliated to Wenzhou Medical University, No. 150, Ximen Street, Linhai, 317000 Zhejiang China

**Keywords:** Endocrine system and metabolic diseases, Obesity, Genetic predisposition to disease

## Abstract

In the sulfotransferase (SULT) superfamily, members of the SULT1 family mainly catalyse the sulfonation reaction of phenolic compounds, which is involved in the phase II metabolic detoxification process and plays a key role in endocrine homeostasis. A coding variant rs1059491 in the *SULT1A2* gene has been reported to be associated with childhood obesity. This study aimed to investigate the association of rs1059491 with the risk of obesity and cardiometabolic abnormalities in adults. This case‒control study included 226 normal weight, 168 overweight and 72 obese adults who underwent a health examination in Taizhou, China. Genotyping of rs1059491 was performed by Sanger sequencing in exon 7 of the *SULT1A2* coding region. Chi-squared tests, one-way ANOVA, and logistic regression models were applied. The minor allele frequencies of rs1059491 in the overweight combined with obesity and control groups were 0.0292 and 0.0686, respectively. No differences in weight and body mass index were detected between the TT genotype and GT + GG genotype under the dominant model, but the levels of serum triglycerides were significantly lower in G-allele carriers than in non-G-allele carriers (1.02 (0.74–1.32) vs. 1.35 (0.83–2.13) mmol/L, *P* = 0.011). The GT + GG genotype of rs1059491 versus the TT genotype reduced the risk of overweight and obesity by 54% (OR 0.46, 95% CI 0.22–0.96, *P* = 0.037) after adjusting for sex and age. Similar results were observed for hypertriglyceridaemia (OR 0.25, 95% CI 0.08–0.74, *P* = 0.013) and dyslipidaemia (OR 0.37, 95% CI 0.17–0.83, *P* = 0.015). However, these associations disappeared after correction for multiple tests. This study revealed that the coding variant rs1059491 is nominally associated with a decreased risk of obesity and dyslipidaemia in southern Chinese adults. The findings will be validated in larger studies including more detailed information on genetic background, lifestyle and weight change with age.

## Introduction

Obesity is a complex and multifactorial chronic metabolic disease and causes a major public health burden worldwide. According to the World Health Organization (WHO), 39% of adults aged 18 years and over were overweight in 2016, and 13% were obese^1^. It has been shown that Asians have higher body fat than Caucasians at the same body mass index (BMI) and that fat is more likely to accumulate in the abdomen^2,3^. Abdominal obesity rather than peripheral obesity is the most common type of obesity in the Chinese population. It is characterized by visceral adipose tissue distribution and is associated with a higher susceptibility to cardiovascular metabolic risk, including increased blood pressure, dyslipidaemia, and diabetes^4^. In an obesogenic environment, excessive dietary energy intake and reduced physical activity are important factors contributing to the development of obesity, while polygenic inheritance and gene‒environment interactions inevitably affect the susceptibility to common obesity^5^.

The *SULT1A2* (sulfotransferase family, cytosolic, 1A, phenols-preferring, member 2) gene is located at 16p12.1, which is close to 16p11.2, a popular region in obesity research. *SULT1A2* encodes a heat-resistant diphenol sulphate-transferase, a key enzyme in sulfuric acid metabolism that catalyses a variety of hormones, including protein sulfonate hormones, oestrogens, oestrogenic alkylphenol, and 17β-oestradiol secretory hormones^6^. *SULT1A1* and *SULT1A2* belong to the same sulfyltransferase superfamily, are located in the same chromatid, have similar structures and functions, and share substrates. There was also a strong linkage between *SULT1A1* and *SULT1A2*^7^. Animal studies have shown that dietary fat regulates *SULT1A1* gene expression in adipose and liver tissues of obese mice^8^. It has been found that a nonsynonymous mutation rs141581853 (P. Arg 213 His) in the *SULT1A1* gene is associated with human obesity, and this locus is linked to *SULT1A2* rs1059491^9^. In addition, an exome-wide scan showed that the *SULT1A2* gene is highly expressed in healthy liver and may be involved in the pathogenesis of nonalcoholic fatty liver disease^10^. To clarify how *SULT1A2* rs1059491 is associated with obesity, our previous study demonstrated that the possible mechanism is the regulation of obesity-related gene expression at both the transcriptional level and the posttranscriptional level^11^.

A published paper from Germany reported that none of the variants in *SULT1A2* were associated with obesity in either children or adults^12^. Furthermore, we previously demonstrated that the coding variant rs1059491 (c.704 T > G) in *SULT1A2* was associated with a high risk of childhood obesity and dyslipidaemia at the genome-wide level in Northern China^11^. However, common obesity is mostly a complex disease with genetic heterogeneity and racial variability. Moreover, the genetic effect of obesity in childhood is usually stronger than that in adults due to the cumulative effect of the life environment. Thus, obesity-associated loci identified in northern children must be replicated in adult populations or in other children. To date, whether *SULT1A2* rs1059491 is associated with adulthood obesity and related cardiometabolic risk factors has not been reported in China. In this study, we aimed to evaluate the relationship between rs1059491 and the risk of obesity and related metabolic disorders in adults.

## Methods

### Study population

We consecutively enrolled adults who underwent weight management from August 10, 2018, to November 30, 2019, and staff health examinations from January 1 to March 10, 2020, in the Health Management Center of our hospital. Those who were pregnant or lactating, were taking medication known to affect body weight or energy metabolism, or had mental illness, bulimia, anorexia, gastrointestinal disorders or morbid obesity were excluded. We designed an unmatched case‒control study of 466 adults including 226 normal-weight, 168 overweight and 72 obese adults, who provided data on anthropometry and venous blood samples. All participants were independent and unrelated individuals. We obtained the informed consent from each participant. This study was approved by the Ethics Committee of Taizhou Hospital of Zhejiang Province in China. All participants’ identity information was anonymous. All detailed protocols followed the principles of our institutional research ethics committee and were in accordance with the Declaration of Helsinki.

### Measurements

Anthropometric data were collected by trained health technicians using a standard protocol. All instruments were validated following the standard methods of the manufacturers. Weight was measured to the nearest 0.1 kg on a balance beam scale, and height was measured to the nearest 0.1 cm with a wall-mounted stadiometer. BMI was calculated as the weight in kilograms divided by the square of the height in metres. Obesity was defined by a BMI ≥ 30 kg/m^2^, and overweight was defined as 25 kg/m^2^ ≤ BMI < 30 kg/m^2^ for adults according to the WHO.

Blood pressure was measured on the right upper arm using an electronic sphygmomanometer (Omron, HBP-9021) with the cuff maintained at heart level after 5 min of rest in a sitting position. Elevated blood pressure was considered with systolic blood pressure (SBP) ≥ 130 mmHg or diastolic blood pressure (DBP) ≥ 85 mmHg or reported use of antihypertensive medication.

### Blood specimen collection and biochemical index detection

Approximately 3 mL of peripheral venous blood was collected from subjects that fasted for 12 h in the morning. After nonanticoagulant centrifugation, serum samples were collected for biochemical tests, and blood clots containing leukocytes were left at the bottom precipitation and stored at − 80 °C for DNA extraction. Fasting plasma glucose (FPG) was measured by the hexokinase method. Serum total cholesterol (TC), low-density lipoprotein cholesterol (LDL-C), high-density lipoprotein cholesterol (HDL-C), and triglyceride (TG) were detected by enzyme-coupled colorimetry. All reagents were purchased and blood biochemical indices were determined by an AU5800 automatic biochemical analyser (BECKMAN COULTER). All laboratory equipment was calibrated.

According to 2016 Chinese guidelines for the management of dyslipidaemia in adults^13^, dyslipidaemia was defined by the presence of one or more of the following component conditions: TC ≥ 5.20 mmol/L (200 mg/dL), LDL-C ≥ 3.40 mmol/L (130 mg/dL), HDL-C < 1.00 mmol/L (40 mg/dL), TG ≥ 1.70 mmol/L (150 mg/dL), or if they were taking anti-dyslipidaemia medication. Impaired fasting glucose (IFG) was diagnosed with FPG ≥ 5.60 mmol/L (100 mg/dL). Abnormalities in either blood pressure, lipids or FPG were considered cardiometabolic abnormalities.

### DNA extraction, polymerase chain reaction (PCR) and sequencing for *SULT1A2* rs1059491

In this study, we collected only one blood sample tube, which needed to be used simultaneously for biochemical testing, serum protein testing and DNA extraction. The genomic DNA of peripheral blood mainly comes from leukocytes according to the TIANamp N96 Blood DNA Kit (Nano MagBio, Cat. no. 4992450) according to the manufacturer’s instructions and then stored at − 20 °C for PCR analysis. The reaction mixture included with 15 µl 2 × Taq PCR Master Mix, 1 µl genomic DNA, 12 µl ddH2O, 1 µl forwards primer and reverse primer (10 pmol/µl), respectively. The final total volume reached 30 µl. The sequences for the PCR primers are presented in Supplementary Table 1, and the expected size was 920 bp. DNA was amplified under the conditions of preheating at 95 °C for 5 min for initial denaturation, followed by 35 cycles of 95 °C for 30 s, 60 °C for 30 s, 72 °C for 45 s, and a final extension at 72 °C for 5 min. Sanger sequencing is the international gold standard for genetic tests and consists of four separate reaction systems, including the target fragment, four deoxyribonucleotides (dNTP), DNA polymerase, and sequencing primers (Supplementary Table 1). The amplified products were sequenced by Sanger sequencing on an ABI 3730xl DNA Analyser, and the sequencing results were analysed by using DNAman (Lynnon Biosoft, America) and Chromas (Technelysium, Australia) software. Genotyping of all samples was performed simultaneously in the same laboratory.

### Statistical analysis

We calculated the sample size and power for an unmatched case‒control study using Quanto 1.2.4 software http://hydra.usc.edu/gxe/.Assuming a dominant genetic model, an allele frequency of 0.06, and overweight and obesity prevalence of 50% in the adults of China, we estimated that an enrolment target of 466 participants (233 per group) would provide the study with greater than 80% statistical power to detect a significant odds ratio of 2.3 for risk of overweight and obesity between the different genotype groups at a significance level of 0.05 using a two-tailed test. The anticipated effect size for the risk of obesity was determined based on previous data obtained from northern Chinese children and adolescents^11^.

Continuous variables are expressed as the mean ± standard deviation (SD). Comparison of variables between groups was performed using one way ANOVA for continuous variables and chi-square tests for categorical variables. Serum TG levels without a normal distribution were described as the geometric mean (interquartile range) and transformed by natural logarithm prior to one-way ANOVA. Hardy–Weinberg equilibrium was performed using the chi-squared test. Assuming a dominant inheritance mode, multivariable logistic regression models were applied to estimate the association between genotype of rs1059491 and overweight combined obesity and related cardiometabolic abnormalities. Sex, age and weight status were adjusted as covariables. The false discovery rate (FDR) approach was used to correct for multiple comparisons. FDR analysis was applied for 18 outcome measures (10 quantitative traits in Table 2, 8 qualitative traits in Table 3) and one variant simultaneously (number of tests: 18 × 1 = 18). In brief, the original *P* value was considered statistically significant only if it was less than the *P* value for FDR. All statistical analyses were performed using SPSS Statistics software version 26.0 (IBM Corp., Armonk, New York). Statistical significance was inferred at a two-tailed *P* value less than 0.05.

### Ethical statement

This study involving human participants was reviewed and approved by the Ethics Committee of Taizhou Hospital of Zhejiang Province in China. The studies involving human participants were reviewed and approved by the Ethics Committee of Taizhou Hospital of Zhejiang Province in China. All procedures were performed in accordance with the guidelines of the institutional ethics committee of the authors and adhered to the tenets of the Declaration of Helsinki. All data, including demographic, biochemical, and genetic information, were anonymized. Personal information is not involved in this article.

## Results

### Clinical characteristics of participants

**Figure 1:**
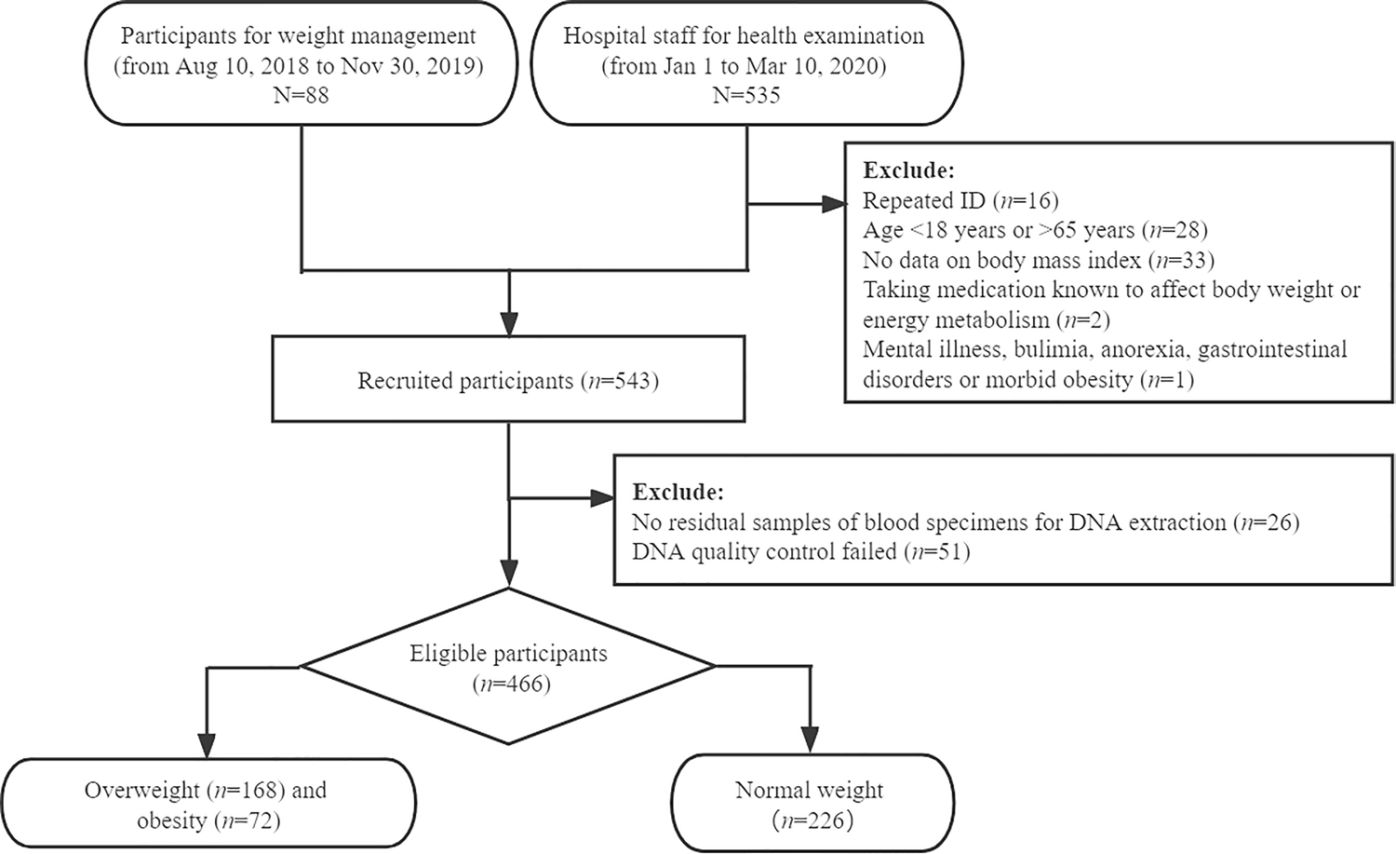
The flowchart of inclusion and exclusion criteria for study participants.

**Figure 2:**
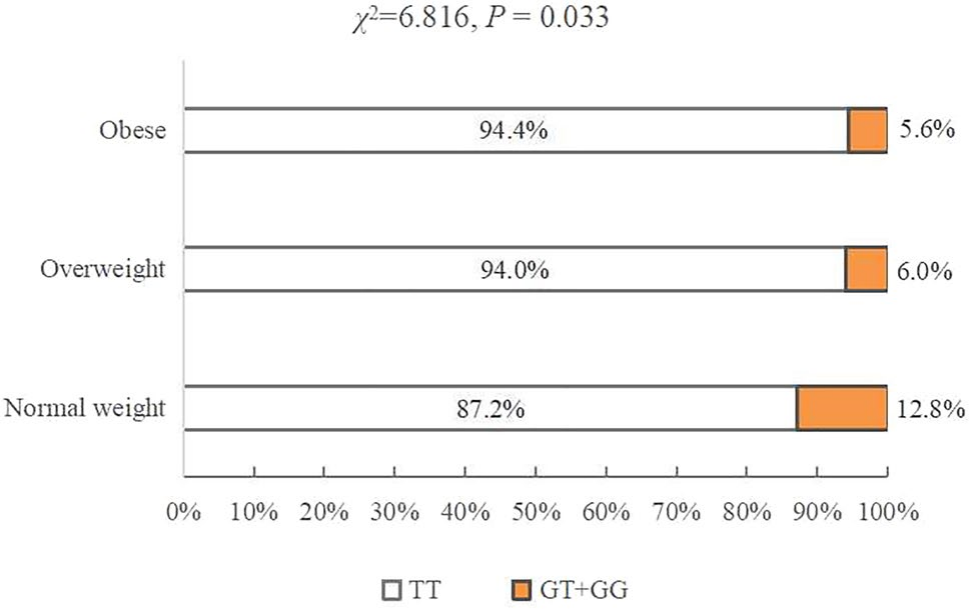
Distribution of genotype of rs1059491 among normal weight, overweight and obese groups.

Individual inclusion and exclusion criteria at each stage of the study are presented in the flowchart (Fig. 1), and a total of 466 participants who provided complete data on anthropometry and venous blood samples were classified as normal weight (226), overweight (168) and obese (72).The general characteristics of the participants in the normal weight group and overweight combined with obesity group are shown in Table 1. The average age (40.6 ± 13.1 years vs. 35.7 ± 9.7 years,* P* < 0.001) and proportion of males (male: 51.7% vs. 16.4%, *P* < 0.001) were higher in the overweight combined obesity group than in the normal weight group. Figure 2 shows the distribution of the genotype of rs1059491 among the normal weight, overweight and obese groups. The frequency of the GT + GG genotype in the overweight and obesity groups was lower than that in the normal weight group (*χ*^2^ = 6.818, *P* = 0.033). In other words, the prevalence of overweight (23.3% vs. 37.4%) and obesity (9.3% vs. 16.1%) was significantly lower in the GT + GG genotype group than in the TT genotype group (*P* = 0.033).

**Table 1:** Clinical characteristics of subjects.

Variables	All	Normal weight	Overweight combined obesity (*n* = 240, 51.5%)	*P*
	(*n* = 466)	(*n* = 226, 48.5%)		
Male, *n* (%)	161 (34.5)	37 (16.4)	124 (51.7)	** < 0.001**
Age (years)	38.2 ± 11.8	35.7 ± 9.7	40.6 ± 13.1	** < 0.001**
Height (cm)	162.8 ± 8.2	161.0 ± 6.9	164.4 ± 9.0	0.226
Weight (kg)	68.2 ± 16.3	56.3 ± 7.6	79.4 ± 14.1	** < 0.001**
BMI (kg/m^2^)	25.5 ± 4.6	21.7 ± 2.1	28.9 ± 3.0	** < 0.001**
SBP (mmHg)	125 ± 16	120 ± 13	130 ± 15	** < 0.001**
DBP (mmHg)	75 ± 12	70 ± 10	79 ± 12	** < 0.001**
FPG (mmol/L)	5.43 ± 1.26	5.25 ± 0.46	5.61 ± 1.67	**0.006**
TG (mmol/L)*	1.31 (0.82–2.06)	1.04 (0.68–1.24)	1.64 (1.21–2.60)	** < 0.001**
TC (mmol/L)	4.84 ± 0.92	4.67 ± 0.92	5.01 ± 0.90	**0.020**
HDL-C (mmol/L)	1.41 ± 0.34	1.57 ± 0.33	1.25 ± 0.26	** < 0.001**
LDL-C (mmol/L)	2.56 ± 0.70	2.38 ± 0.66	2.74 ± 0.69	** < 0.001**

Values are expressed as the mean ± standard deviation (SD).

Triglycerides were skewed distribution, expressed as a geometric mean (interquartile range) and one way ANOVA was performed after logarithmic transformation.

Overweight was defined as 25 kg/m^2^ ≤ BMI < 30 kg/m^2^ and obesity as BMI ≥ 30 kg/m^2^.

Bold indicates *P* value < 0.05.

BMI, body mass index; SBP, systolic blood pressure; DBP, diastolic blood pressure; FPG, fasting plasma glucose; TG, triglyceride;TC, total cholesterol; HDL-C, high-density lipoprotein cholesterol; LDL-C, low-density lipoprotein cholesterol.

### Effect of the rs1059491 genotype on obesity-related quantitative traits

As shown in Table 2, the levels of serum triglycerides (1.02 (0.74–1.32) vs. 1.35 (0.83–2.13) mmol/L, *P* = 0.011) was significantly lower in the GT + GG genotype of rs1059491 than in the TT genotype. Similarly, BMI levels were nominally reduced in G-allele carriers compared to their counterparts (24.3 ± 4.5 vs. 25.7 ± 4.6 kg/m^2^, *P* = 0.063). No significant differences were observed in other indices between the different genotype groups. Under a dominant genetic model, carrying the G allele of rs1059491 nominally decreased serum TG levels even after adjustment for age and sex (*β* = -1.23 mmol/L, *P* = 0.031). However, the associations disappeared after correction for multiple tests. Moreover, there was no significant influence of the genotype of *SULT1A2* rs1059491 on obesity-related phenotypic traits, including weight, SBP, DBP, TC, HDL-C, LDL-C, and FPG.

**Table 2:** Effect of GT + GG genotype versus TT genotype of rs1059491 under dominant model on obesity-related phenotypic traits.

Variables	Genotype of rs1059491		Model 1			Model 2		
	TT (*n* = 423)	GT + GG (*n* = 43)	*β*	95%*CI*	*P*	*β*	95%*CI*	*P*
Height (cm)	163.2 ± 8.2	162.4 ± 8.5	− 0.80	− 3.66 to 2.05	0.580	0.54	− 1.39 to 2.47	0.585
Weight (kg)	68.6 ± 16.3	64.4 ± 15.5	− 4.22	− 9.33 to 0.89	0.105	− 1.99	− 5.86 to 1.88	0.313
BMI (kg/m^2^)	25.7 ± 4.6	24.3 ± 4.5	− 1.36	− 2.80 to 0.08	0.063	− 0.83	− 2.11 to 0.46	0.205
SBP (mmHg)	125 ± 16	126 ± 12	1.29	− 3.66 to 6.23	0.610	3.75	− 0.64 to 8.13	0.094
DBP (mmHg)	75 ± 12	74 ± 10	− 0.89	− 4.62 to 2.83	0.638	0.71	− 2.66 to 4.07	0.681
FPG (mmol/L)	5.46 ± 1.31	5.07 ± 0.40	− 0.39	− 0.82 to 0.04	0.073	− 0.31	− 0.72 to 0.10	0.137
TG (mmol/L)	1.35 (0.83 ‒ 2.13)	1.02 (0.74 ‒ 1.32)	− 1.32	− 1.64 to (− 1.07)	**0.011**	− 1.23	− 1.49 ‒to (− 1.02)	**0.031**
TC (mmol/L)	4.85 ± 0.93	4.76 ± 0.92	− 0.09	− 0.41 to 0.22	0.556	− 0.04	− 0.35 to 0.27	0.807
HDL-C (mmol/L)	1.40 ± 0.34	1.47 ± 0.32	0.07	− 0.04 to 0.19	0.229	0.04	− 0.06 to 0.15	0.425
LDL-C (mmol/L)	2.57 ± 0.70	2.51 ± 0.76	− 0.06	− 0.30 to 0.18	0.638	− 0.01	−0.24 to 0.22	0.941

Model 1: Unadjusted.

Model 2: Adjusted for sex and age.

Bold indicates *P* value < 0.05.

BMI, body mass index; SBP, systolic blood pressure; DBP, diastolic blood pressure; FPG, fasting plasma glucose; TG, triglyceride; TC, total cholesterol; HDL-C, high-density lipoprotein cholesterol; LDL-C, low-density lipoprotein cholesterol.

### The association of the rs1059491 genotype with cardiometabolic abnormalities

The ancestral allele was T, allele G was the minor allele of rs1059491, and the overall minor allele frequency (MAF) was 0.048. The MAF in the overweight and obesity group was lower than that in the control group (0.0292 vs. 0.0686). The genotype and allele frequencies of rs1059491 were in accordance with Hardy–Weinberg equilibrium in the control groups for each qualitative trait (all *P* > 0.05, Supplementary Table 2). Table 3 displays the associations of the rs1059491 genotype with the risk of being overweight combined with obesity and other cardiometabolic abnormalities after adjusting for age and sex assuming a dominant genetic model. Individuals carrying the minor allele G of rs1059491 had a lower risk of overweight and obesity than non-G-allele carriers (OR 0.46, 95% CI 0.22–0.96, *P* = 0.037). Similar results were also observed for hypertriglyceridaemia (OR 0.28, 95% CI 0.09–0.88, *P* = 0.030) and dyslipidaemia (OR 0.41, 95% CI 0.18–0.93, *P* = 0.032) even after adjusting for sex, age and weight status. These significant associations were abolished by further adjustment for multiple testing. However, no associations were observed between the rs1059491 genotype and the risk of elevated blood pressure, impaired fasting glucose, elevated TC, elevated LDL-C, and decreased HDL-C.

**Table 3:** Effect of rs1059491 genotype on cardiometabolic abnormalities under dominant model (GT + GG versus TT).

Cardiometabolic abnormality	Model 1			Model 2			Model 3		
	*OR*	95%*CI*	*P*	*OR*	95%*CI*	*P*	*OR*	95%*CI*	*P*
Overweight combined obesity (BMI ≥ 25 kg/m^2^)	0.42	0.22‒0.82	**0.011**	0.46	0.22‒0.96	**0.037**	–	**–**	**–**
Elevated blood pressure (SBP/DBP ≥ 130/85 mmHg)	1.19	0.63‒2.27	0.591	1.67	0.83‒3.36	0.149	2.13	1.01‒4.48	**0.046**
Impaired fasting glucose (FPG ≥ 5.60 mmol/L (100 mg/dL))	0.29	0.09‒0.98	**0.047**	0.33	0.09‒1.14	**0.080**	0.34	0.10‒1.21	0.097
Elevated triglycerides (fasting triglycerides ≥ 1.7 mmol/L (150 mg/dL))	0.24	0.08‒0.69	**0.008**	0.25	0.08‒0.74	**0.013**	0.28	0.09‒0.88	**0.030**
Elevated TC (fasting TC ≥ 5.2 mmol/L (200 mg/dL))	0.71	0.33‒1.52	0.379	0.79	0.36‒1.72	0.552	0.83	0.38 ‒ 1.82	0.635
Decreased HDL-C (HDL-C < 1.0 mmol/L (40 mg/dL))	0.64	0.15‒2.79	0.552	0.78	0.17‒3.54	0.742	0.96	0.20‒4.69	0.959
Elevated LDL-C (fasting LDL-C ≥ 3.40 mmol/L (130 mg/dL))	0.96	0.32‒2.85	0.942	1.04	0.35‒3.13	0.940	1.15	0.38‒3.49	0.809
Dyslipidaemia	0.35	0.17‒0.75	**0.007**	0.37	0.17‒0.83	**0.015**	0.41	0.18‒0.93	**0.032**

Model 1: Unadjusted.

Model 2: Adjusted for sex and age.

Significant are in value [bold].

Model 3: Adjusted for sex, age and weight status (normal weight, overweight or obese).

BMI, body mass index; SBP, systolic blood pressure; DBP, diastolic blood pressure; FPG, fasting plasma glucose; TC, total cholesterol; HDL-C, high-density lipoprotein cholesterol; LDL-C, low-density lipoprotein cholesterol.

Dyslipidaemia was defined by the presence of one or more of the followingcomponents conditions: TC ≥ 5.20 mmol/L (200 mg/dL), LDL-C ≥ 3.40 mmol/L (130 mg/dL), HDL-C < 1.00 mmol/L (40 mg/dL), TG ≥ 1.70 mmol/L (150 mg/dL), or if they were taking anti-dyslipidaemia medication.

## Discussion

The mutation frequency of *SULT1A2*rs1059491 (c.704 T/G, p.Asn235Thr) varies greatly among different populations^14^. In our study population, the MAF of rs1059491 was only 0.048, and almost all Gallele carriers were heterozygous. Our results are similar to the Gallele frequency of the EastAsian population in the Genome Aggregation Database (gnomAD) (0.0553) and ALFA (Allele Frequency Aggregator) projects (0.054)^15^.

To our knowledge, the present study is the first to investigate the genetic association of the *SULT1A2* rs1059491 variant with overweight and obesity in southern Chinese adults who underwent a physical examination. The genotyping results revealed that the frequency of the minor allele G was lower in the obesity or cardiometabolic abnormality group than in their counterpart. The variant rs1059491 G-allele significantly decreased the risk of overweight and obesity, hypertriglyceridaemia and dyslipidaemia in adults after adjusting for sex and age. However, our previous study indicated that the G allele is a risk factor for obesity in children from northern China^11^. This inconsistency may be due to age and regional differences between the two populations. Genetic contribution to BMI varies with age and may be greater during childhood than adulthood because of the cumulative influence of environmental factors^16^. Therefore, long-term follow-up is required to monitor the occurrence and development of obesity and related metabolic abnormalities to further verify the effect of genes.

Rs1059491 is a missense variant in exon 7 of *SULT1A2*. Different software predictions show that rs1059491: p.Asn235Thr (c.704 T/G) is related to gene function (‘probably damaging’ PolyPhen-2, ‘nonneutral’ SNAP and ‘deleterious’ SIFT). However, our previous findings suggest that rs1059491 is an eQTL that may contribute to obesity by altering the expression of obesity-related genes rather than affecting protein structure^11^. Available transcriptome and GETxdata revealed that rs1059491 was located at transcription factor-binding sites of PPARγ2 and RXRA^17^, which could lead to abnormal lipid metabolism and obesity.

For the first time, we found an association between the coding variant rs1059491 in the *SULT1A2* gene and obesity risk in adults, which laid a foundation for further research on gene function and the molecular mechanism of obesity. Previous fine mapping studies of obesity candidate genes in the 16p11.2 region indicated that rare mutations in nearby genes *APOBR*^12^ and *SH2B1*^18^ but not *SULT1A2* were strongly associated with the risk of obesity or extreme obesity. However, a genome-wide association study reported an important BMI-related SNP rs7359397 located in *SH2B1*, which was highly linked with the nearby nonsynonymous variant rs1059491 of *SULT1A2* (R^2^ > 0.75)^19^. *SH2B1* is the most likely obesity-causing gene in this region^20^. The *SH2B1* protein is involved in the regulation of energy balance by increasing leptin and insulin in the downstream signalling pathway.

In addition, a study of 230 breast cancer patients in Taiwan found that *SULT1A2* gene polymorphism may be associated with the early onset of breast cancer patients^21^. Both breast cancer and obesity are associated with increased levels of *SULT1A2* substrate 17β-oestradiol, estrone and estrone sulphate^22^. Recently, another study showed that BMI is the only breast cancer risk factor that affects metabolic fluxes to adducts of oestrogens with DNA (via congruent adverse influence on levels of oestrogens, *CYP1B1* and *SULT1A2*)^23^. These results suggest that *SULT1A2* may be involved in the regulation of body weight and energy balance by regulating steroid metabolism^24^. The indirect evidence supported the association of the coding variantrs1059491 in *SULT1A2* with the risk of obesity and dyslipidaemia.

There are several limitations in our study. First, the power calculation was performed, and our study had 75% power (enrolment of 240 overweight and obese subjects) to detect ORs of 0.42 for overweight and obesity under a dominant genetic model. Although we found a nominal association of rs1059491 with overweight and obese individuals, the association was no longer significant after correction for multiple testing, so further validation studies with a larger sample size are warranted. Second, the individuals’ lifestyles, such as smoking, drinking, diet, and exercise, have not been considered. Third, this study was a case‒control design. The phenotypic data collection was only done once, so we did not observe how the weight status changes over time. Fourth, our study only included participants from one medical centre in China, most of whom were medical personnel with high work intensity and stress, which may cause selection bias. In addition, we did not investigate whether our study subjects came from other regions with different genetic backgrounds. However, our study site was in a county in southern China, where Han Chinese populations are concentrated. Population stratification may not be a concern. Future studies with larger sample sizes should be validated in various populations with different ages and geographic regions. Therefore, the results of this study may not be generalizeable or widely applicable to other populations.

## Conclusion

In conclusion, this study revealed that the coding variant rs1059491 in the *SULT1A2* gene is nominally associated with a decreased risk of obesity and dyslipidaemia in southern Chinese adults. The findings will be validated in larger studies that include more detailed information on genetic background, lifestyle and weight change with age.

## Supplementary Information

Supplementary Information 1.

Supplementary Information 2.

## Data Availability

All data generated or analysed during this study are included in this published article and its supplementary information files.
