# Author Correction: Association of *SULT1A2* rs1059491 with obesity and dyslipidaemia in southern Chinese adults

**DOI:** 10.1038/s41598-023-35762-9

**Published:** 2023-05-31

**Authors:** Hai‑Yan Lv, Guifeng Shi, Cai Li, Ya‑Fei Ye, Ya‑Hong Chen, Li‑Hua Chen, Tao‑Hsin Tung, Meixian Zhang

**Affiliations:** 1 Department of Clinical Laboratory, Taizhou Hospital of Zhejiang Province Affiliated to Wenzhou Medical University, Linhai, 317000 Zhejiang China; 2 Department of Preventive Health Care, Taizhou Hospital of Zhejiang Province Affiliated to Wenzhou Medical University, Linhai, 317000 Zhejiang China; 3 Department of Neurology, Taizhou Hospital of Zhejiang Province Affiliated to Wenzhou Medical University, Linhai, 317000 Zhejiang China; 4 Health Management Centre, Taizhou Hospital of Zhejiang Province Affiliated to Wenzhou Medical University, Linhai, 317000 Zhejiang China; 5 Public Scientific Research Platform, Taizhou Hospital of Zhejiang Province Affiliated to Wenzhou Medical University, Linhai, 317000 Zhejiang China; 6 Evidence-Based Medicine Center, Taizhou Hospital of Zhejiang Province Affiliated to Wenzhou Medical University, No. 150, Ximen Street, Linhai, 317000 Zhejiang China

Correction to: *Scientific Reports* https://doi.org/10.1038/s41598-023-34296-4, published online 04 May 2023

The Acknowledgments section in the original version of this Article was omitted. The Acknowledgments section now reads:

“This work was supported by the Zhejiang Basic Public Welfare Research Project (LGF20H260013), the Initial Scientific Research Fund for PhD from Taizhou Hospital of Zhejiang Province (2018BSKYQDJJ15) and the Taizhou Science and Technology Planning Project (1802ky01).”

The original Article has been corrected.

