# Supplementary material for: Association of SULT1A2 rs1059491 with obesity and dyslipidaemia in southern Chinese adults
Source: Sci Rep. 2023 May 4;13:7256. doi: 10.1038/s41598-023-34296-4 (PMC10160091; doi:10.1038/s41598-023-34296-4)
Supplement: Supplementary file 2 — Supplementary Information 2. [file 41598_2023_34296_MOESM2_ESM.docx]

**Association of *SULT1A2* rs1059491 with obesity and dyslipidaemia in southern Chinese adults**

**Supplementary Files**

**Supplementary Table 1** The features of primers used in this study.

| Primer name | Primer sequence | Product size | Annealing temp. °C |
| --- | --- | --- | --- |
| rs1059491-F | TCAATGTGGCTCAGAGATAC | 920bp | 60℃ |
| rs1059491-R | GTCGAGGAGCTGGCTCTATG | 920bp | 60℃ |
| rs1059491-SEQ | TCTTCGCATAGTCCGCATCG | 920bp | 60℃ |
| rs1059491-SEQ | ATCAGCAATCCAAGCCTCCA | 920bp | 60℃ |

**Supplementary Table 2.** Genotype and allele distributions of rs1059491 and Hardy-Weinberg equilibrium test

| **Cardiometabolic abnormality** | **Case** | | |  | **Control** | | |
| --- | --- | --- | --- | --- | --- | --- | --- |
|  | **TT/TG/GG** | **MAF** | **PH-W** |  | **TT/TG/GG** | **MAF** | **PH-W** |
| Overweight combined obesity | 226/14/0 | 0.0292 | 0.642 |  | 197/27/2 | 0.0686 | 0.329 |
| Elevated blood pressure:   SBP/DBP ≥130/85 mmHg | 154/17/1 | 0.0552 | 0.487 |  | 245/23/1 | 0.0465 | 0.564 |
| Impaired fasting glucose:  FPG ≥ 5.60 mmol/L | 84/2/1 | 0.0230 | **<0.001** |  | 272/32/1 | 0.0557 | 0.954 |
| Elevated triglycerides:   fasting triglycerides ≥1.7 mmol/L | 122/4/0 | 0.0159 | 0.856 |  | 234/30/2 | 0.0639 | 0.349 |
| Elevated TC:  fasting TC ≥5.2 mmol/L | 125/9/1 | 0.0407 | 0.087 |  | 231/25/1 | 0.0525 | 0.715 |
| Decreased HDL-C:  HDL-C <1.0 mmol/L | 30/2/0 | 0.0313 | 0.855 |  | 326/32/2 | 0.0500 | 0.222 |
| Elevated LDL-C:  fasting LDL-C ≥3.4 mmol/L | 41/3/1 | 0.0556 | **0.014** |  | 315/31/1 | 0.0476 | 0.798 |
| Dyslipidaemia | 186/9/1 | 0.0281 | **0.027** |  | 170/25/1 | 0.0689 | 0.938 |

Overweight was defined as 25 kg/m^2^ ≤ body mass index < 30 kg/m^2^ and obesity as body mass index ≥ 30 kg/m^2^.

Dyslipidaemia was defined by the presence of one or more of the following components conditions: TC ≥ 5.20 mmol/L (200 mg/dL), LDL-C ≥ 3.40 mmol/L (130 mg/dL), HDL-C < 1.00 mmol/L (40 mg/dL), TG ≥ 1.70 mmol/L (150 mg/dL), or if they were taking anti-dyslipidaemia medication.

MAF, minor allele frequency; SBP, systolic blood pressure; DBP, diastolic blood pressure; TC, total cholesterol; HDL-C, high-density lipoprotein cholesterol; LDL-C, low-density lipoprotein cholesterol.
